# A novel power-driven fractional accumulated grey model and its application in forecasting wind energy consumption of China

**DOI:** 10.1371/journal.pone.0225362

**Published:** 2019-12-05

**Authors:** Peng Zhang, Xin Ma, Kun She

**Affiliations:** 1 School of Information and Software Engineering, University of Electronic Science and Technology of China, Chengdu, China; 2 School of Science, Southwest University of Science and Technology, Mianyang, China; 3 State Key Laboratory of Oil and Gas Reservoir Geology and Exploitation, Southwest Petroleum University, Chengdu, China; Huazhong University of Science and Technology, CHINA

## Abstract

Wind energy is one of the most important renewable resources and plays a vital role in reducing carbon emission and solving global warming problem. Every country has made a corresponding energy policy to stimulate wind energy industry development based on wind energy production, consumption, and distribution. In this paper, we focus on forecasting wind energy consumption from a macro perspective. A novel power-driven fractional accumulated grey model (PFAGM) is proposed to solve the wind energy consumption prediction problem with historic annual consumption of the past ten years. PFAGM model optimizes the grey input of the classic fractional grey model with an exponential term of time. For boosting prediction performance, a heuristic intelligent algorithm WOA is used to search the optimal order of PFAGM model. Its linear parameters are estimated by using the least-square method. Then validation experiments on real-life data sets have been conducted to verify the superior prediction accuracy of PFAGM model compared with other three well-known grey models. Finally, the PFAGM model is applied to predict China’s wind energy consumption in the next three years.

## Introduction

Wind energy is one of the important vital resources of renewable energy, which is widely distributed with large reserves. Wind energy and other renewables will play a vitally important role in solving global warming issues and reducing carbon emission in future decades. International energy agency (IEA) has optimistically estimated that renewables will account for 39% share of total electricity generation by 2050. In light of Global Wind Report 2018 of the GWEC, it noticed that new wind installed capacity had overtaken new fossil fuel capacity for the first time in many developing or mature markets. The global total cumulative wind installed capacity has brought up to 591GW at the end of 2018 with new installations 51.3GW. In China, the total wind installed capacity has reached 211GW in 2018, which indicates that the wind energy target of Five-Year-Plan 2016-2020 has been achieved two years ahead of schedule. Along with the rapid development of the energy industry, it also brings a great challenge to make energy policy upgrade energy structure. According to the definition in the literature [1], the energy policy of an entity (especially a government) is used to solve the problems of energy production, consumption, and distribution [2]. Most scholars mainly focused on forecasting the state of wind energy, including wind speed forecasting and wind power forecasting, which is expected to provide a reference for formulating energy production planning from micro-perspective. These forecasting approaches mainly include deterministic forecasting and uncertainty analysis [3]. For these approaches, forecasting wind energy is considered as a prediction process of stochastic time series. Usually, the future values are predicted by these data-driven algorithms based on the historical wind speed or wind energy sequence or other related data. Many scholars applied Artificial Neural Network (ANN) to forecast wind energy. Numerous variants of ANN, such as Back Propagation Neural Network (BPNN) [4, 5], Radial Basis Function Neural Network (RBFNN) [6], Generalized Regression Neural Network (GRNN) [7] and Wavelet Neural Network (WNN) [8, 9], were also proposed to predict the future wind energy. Besides, some scholars utilized Support Vector Machine (SVM) [10] or its variants [11, 12] to forecast wind energy. Deep learning approaches, such as Autoencoder (AE), Deep Boltzmann Machine (DBM) [13], Convolutional Neural Network (CNN) [14], Recurrent Neural Networks (RNNs) [15] and so on, were also adopted to forecast the future wind energy. Though these approaches can be used to forecasting wind energy, they usually need a larger dataset, including historical data and some exogenous data, to train the predictors for better prediction performance. And they were often used to predict hours or minutes ahead of wind energy or speed for making the production plan of a company or energy farm in a local region [16, 17]. Besides, many scholars utilized ARMA, ARIMA, and their successors to predict wind energy or wind speed [18, 19]. However, the study of wind energy consumption prediction from the macro view is very few at present. Previously, many scholars mainly concentrated in study of forecasting electricity [20–29], natural gas [30, 31], oil [32, 33], nuclear [34, 35], renewable [36, 37] energy consumption and so on. Forecasting wind energy consumption is also an important task for making energy policy and plan for the government. Grey prediction model is an effective prediction approach, which is one of the best choices for solving the wind energy consumption prediction problem with small samples.

Recently, scholars have conducted numerous studies to improve prediction performance and enlarge the application range of grey models. Lots of novel or improved grey models have been put forward to solve prediction problems with partially know or unknown information effectively. On the one hand, many scholars devoted their efforts to optimize the initial GM(1,1) model. Wang et al. proposed a rolling grey model with PSO and data cleaning techniques to predict Beijing’s tertiary industry effectively [38]. Xia et al. [39] proposed an improved grey model with grey input of time power based on the new information priority. Ding et al. overcame the fixed structure and poor adaptability of GM(1,1) model and put forward an optimized grey model called NOGM(1,1) to forecast China’s electricity consumption [20]. Xu et al. proposed IRGM(1,1) model with optimizing the initial condition of time response function [21]. Wang et al. proposed SGM(1,1) model to forecast the seasonal time series [23]. Besides, other improved grey models such as WBGM(1,1) [40], GARGM(1,1) [41], Nash NBGM [42], SIGM [43], GRA-IGSA [44] and so on were proposed and obtained satisfactory prediction accuracy. But these models have the same insufficiency of traditional GM(1,1) model because they are essentially grey models with one order and one variable. For the multi-variable time series, Wang et al [45] proposed an improved GM(1,n) model which considered the effects of the dependent variables. Zeng et al. improved the GM(1,n) model with a dynamic background parameter [46]. Soon afterwards Zeng et al. [47] presented a new multi-variable grey model to enhance the structure compatibility of grey model. Ma et al. [48] proposed a parameter optimization method for CGM(1,n) model to promote its prediction accuracy. These efforts not only enriched the theory of grey system but also improved the prediction performance of grey prediction model.

On the other hand, fractional order accumulating operator was imported to decrease the stochasticity and uncertainty of raw sequence to boost the performance of grey model. Wu et al. initially improved traditional GM(1,1) with fractional order accumulated operator called FAGM(1,1), which guarantees the priority of new information under small fractional order and boosts the prediction accuracy compared with traditional GM(1,1) model [49]. Yang et al. generalized the traditional GM(1,1) models with fractional calculus of which fractional-order derivative is profoundly determined the accuracy of prediction and can be optimized by intelligent algorithms [27]. Mao et al. proposed a fractional grey model based on fractional order derivative called FGM(q,1) of which the whitening equation is a fractional order differential equation, and forecasted the gross national income per capita accurately [50]. Some researchers optimized the grey action quantity of FAGM(1,1) and achieved better prediction accuracy. Ma et al. optimized the grey input of the original FAGM(1,1) model with a fractional time delay term and applied it to predict the gas consumption and coal consumption [51]. Wu et al. proposed a fractional FAGM(1,1,k) model with linear grey input of time instead of constant grey input in initial FAGM(1,1) model and optimized it with optimal linear parameters and optimal order [34]. Then Wu et al. proposed a fractional accumulated Bernoulli grey model and adopted an intelligent optimization algorithm to seek optimal fractional order of this model [37]. Optimization of fractional order accumulation is another improvement for the traditional FAGM(1,1) model. Ma et al. firstly proposed a CFGM(1,1) model in which the computational complexity of accumulation is lower than that of traditional fractional accumulated operator [52]. Zeng proposed a self-adaptive intelligent fractional grey model called as SAIGMFO model and predicted the electricity consumption of China [53]. Besides, the fractional multivariate grey model is another kind of grey model to deal with multivariate time series. Ma et al. proposed a discrete multivariate grey prediction model (FDGM) and mathematically proved that it is an unbiased grey model that was applied in four real-life applications and achieved better accuracy compared with other well-known grey models [54]. Though these fractional grey models have gained better performance, boosting prediction accuracy of fractional grey models is still worth studying.

In this paper, we focus on forecasting wind energy consumption by using grey models to provide reference information for formulating and adjusting energy policy. In order to boost the prediction accuracy, we improved the classic fractional grey model with an exponential grey input term. There are two aspects of the contribution. On the one hand, we proposed a novel power-driven fractional accumulated grey model called PFAGM of which optimal order is sought out by WOA algorithm. Meanwhile, PFAGM model can be easily reduced into the classical grey model and fractional grey model. To some extends, PFAGM model has better adaptability than classical grey models. On the other hand, PFAGM model is applied to forecast China’s wind energy consumption in the next three years.

The rest of this paper is structured as follows. In section 2, we will introduce the fractional order accumulation and the classic fractional accumulated grey model in detail. In section 3, a power-driven fractional accumulated grey model (PFAGM) with optimization of grey action quantity is proposed. In section 4, we validate the prediction accuracy of PFAGM model on several real-life datasets compared with those of three well-known grey models. In section 5, PFAGM model is utilized to forecast the total wind energy consumption of China. In the last section, several conclusions are drawn.

## Fractional accumulated operation and fractional accumulated grey model

The fractional accumulating operation plays a very important role in grey prediction applications. It can be used to decrease the randomness of raw sequence data and boost the performance of grey models. The detail of fractional accumulate operation and its inverse operation are introduced as follows.

### Fractional accumulated operation

For an original data sequence *X*^(0)^ = (*x*^(0)^(1), *x*^(0)^(2), ⋯, *x*^(0)^(*n*)), the *r*-order accumulated operation sequence can be defined as follow.

**Definition 1** *The r-order fractional accumulated operation sequence of raw data is defined as*:

$$\begin{array}{c} X^{\left(r\right)}=(x^{\left(r\right)}\left(1\right),x^{\left(r\right)}\left(2\right),\cdots ,x^{\left(r\right)}\left(n\right)). \end{array}$$ (1)

*where* $x^{\left(r\right)}\left(k\right)=\sum_{i=1}^{k}\frac{(k-i+r-1)!}{(r-1)!(k-i)!}x^{\left(0\right)}\left(i\right),k=1,2,\ldots ,n$.

**Definition 2** *The inverse r-order fractional accumulated operation sequence is defined as*:

$$\begin{array}{c} X^{\left(-r\right)}=(x^{\left(-r\right)}\left(1\right),x^{\left(-r\right)}\left(2\right),\cdots ,x^{\left(-r\right)}\left(n\right)). \end{array}$$ (2)

*where* $x^{\left(-r\right)}\left(k\right)=\sum_{i=1}^{k}\frac{(k-i-r-1)!}{(-r-1)!(k-i)!}x^{\left(0\right)}\left(i\right),k=1,2,\ldots ,n$.

### Methodology of fractional order accumulated grey model

The fractional order accumulated grey model abbreviated as FAGM(1,1) was firstly proposed by Wu et al. in 2013 [49]. The whitening differential equation of FAGM(1,1) model is defined as:

$$\begin{array}{c} \frac{dx^{\left(r\right)}\left(t\right)}{dt}+ax^{\left(r\right)}\left(t\right)=b. \end{array}$$ (3)

where *a* is the development coefficient, *b* is the grey input, and *r* is the fractional order of grey model. Integrating both side of Eq (3) within the interval [*k* − 1, *k*], the discrete form of FAGM model is obtained as follow:

$$\begin{array}{c} x^{\left(r\right)}\left(k\right)-x^{\left(r\right)}\left(k-1\right)+az^{\left(r\right)}\left(k\right)=b. \end{array}$$ (4)

where *z*^(*r*)^(*k*) = 0.5(*x*^(*r*)^(*k*) + *x*^(*r*)^(*k*)).

In order to estimate the parameters of FAGM(1,1) model, the least-square method is used to solve the problem with the objective of minimizing the errors of simulation under the assumption that the order is given. So, the parameters *a* and *b* can be calculated as follow:

$$\begin{array}{c} {\left[a,b\right]}^{T}={\left(A^{T}A\right)}^{-1}A^{T}Y. \end{array}$$ (5)

where

$$\begin{array}{c} Y_{u}=[\begin{array}{c} x^{\left(r\right)}\left(2\right)-x^{\left(r\right)}\left(1\right)\\ x^{\left(r\right)}\left(3\right)-x^{\left(r\right)}\left(2\right)\\ \vdots \\ x^{\left(r\right)}\left(u\right)-x^{\left(r\right)}\left(u\right) \end{array}],A_{u}=[\begin{array}{cc} z^{\left(r\right)}\left(2\right) & 1\\ z^{\left(r\right)}\left(3\right) & 1\\ \vdots & \vdots \\ z^{\left(r\right)}\left(u\right) & 1 \end{array}]. \end{array}$$ (6)

in which *u* is the number of in-samples used to build model. Then we solve the differential Eq (3) and have

$$\begin{array}{c} x^{\left(r\right)}\left(t\right)=Ce^{-at}+\frac{b}{a}. \end{array}$$ (7)

Substituting the initial condition into Eq (7) and setting *t* = *k*, the time response function of FAGM model is obtained as follow:

$$\begin{array}{c} x_{p}^{\left(r\right)}\left(k\right)=(x^{\left(0\right)}\left(1\right)-\frac{b}{a})e^{-a(k-1)}+\frac{b}{a}. \end{array}$$ (8)

where *k* = 2, 3, …, *n*. However, the time response sequence is only an intermediate result and needs to be restored to restored values by using inverse r-order fractional accumulated operation. The restored value can be represented as:

$$\begin{array}{c} x_{p}^{\left(0\right)}\left(k\right)=\sum_{i=1}^{k}\frac{(k-i-r-1)!}{(-r-1)!(k-i)!}x_{p}^{\left(r\right)}\left(i\right). \end{array}$$ (9)

where *k* = 1, 2, …, *n*.

## The proposed fractional accumulated grey model

In this section, we prove that the grey action quantity of fractional grey model built by various subsequences with the same length changes with time in a homogeneous exponential sequence. Then a novel power-driven fractional accumulated grey model is proposed, which optimizes the classical FAGM model with an exponential grey action quantity. It makes the grey action quantity change from a constant term to an exponential term of time.

### The basis of grey action quantity optimization

The optimization of grey input is a remarkable approach to improve classical grey models and promote their prediction accuracy. The basis of grey action quantity optimization is that the grey input of the grey model changes with time in the real-world grey system. Xu et al. [55] presented a theory to illustrate that the grey action quantity of the classical grey model would change with time. The theory is represented as follow:

**Theorem 1** *Assuming that the time series X*^(0)^(*k*) = *Ae*^λ(*k*−1)^, *k* = 1, 2, ⋯ *is raw sequence, there are two subsequence* $X_{1}^{\left(0\right)}\left(k\right)=Ae^{\lambda (k-1)},k=1,2,\ldots ,n$ *and* $X_{2}^{\left(0\right)}\left(k\right)=Ae^{\lambda (k+t-1)},k=1,2,\ldots ,n$ *with the same number of samples. The parameters a*_1_ *and b*_1_ *are respectively the development coefficient and grey input of the GM(1,1) model built by* $X_{1}^{\left(0\right)}$. *The parameters a*_2_ *and b*_2_ *are respectively the development coefficient and grey input of the GM(1,1) model built by* $X_{2}^{\left(0\right)}$. *Then, a*_1_ = *a*_2_ *and b*_2_ = *b*_1_ *e*^λ*t*^.

Theorem 1 indicates that the grey action quantity of GM(1,1) changes with time. If the first order term and constant term of Maclaurin’s series of *e*^λ*t*^ are only remained, the SAIGM(1,1) model is obtained with the grey input term *bt* + *c*. If the first order term of Maclaurin’s series of *e*^λ*t*^ is only remained, the NGM(1,1) model can be obtained with the grey input term *bt*. Obviously, the grey inputs of SAIGM and NGM are linear time functions. In fact, the grey input of the grey system is nonlinear. Notably, the EOGM(1,1) model has been presented when the term *be*^λ*t*^ is directly used as its grey action quantity.

For the fractional accumulation grey model, its grey action quantity should vary with time according to the above-mentioned optimization of the classical GM(1,1) model. This proposition can also be illustrated with the theorem as follow:

**Theorem 2** *Assuming that the sequence Y*^(0)^ = {*y*^(0)^(*k*) = *Ae*^λ(*k*−1)^|*k* = 1, 2, ⋯} *is raw data, there are two subsequence* $Y_{1}^{\left(0\right)}=\{y_{1}^{\left(0\right)}\left(k\right)=Ae^{\lambda (k-1)}|k=1,2,\ldots ,n\}$ *and* $Y_{2}^{\left(0\right)}=\{y_{2}^{\left(0\right)}\left(k\right)=Ae^{\lambda (k+t-1)}|k=1,2,\ldots ,n\}$ *with the same number of samples. The parameters a*_1_ *and b*_1_ *are respectively the development coefficient and grey input of the FAGM(1,1) model built by* $Y_{1}^{\left(0\right)}$. *The parameters a*_2_ *and b*_2_ *are respectively the development coefficient and grey input of the FAGM(1,1) model built by* $Y_{2}^{\left(0\right)}$. *Then, a*_1_ = *a*_2_ *and b*_2_ = *b*_1_ *e*^λ*t*^.

**Proof 1** *According to the modeling procedure of FAGM(1,1) model, the fractional order accumulation generated sequence of* $Y_{1}^{\left(0\right)}$ *is represented as* $Y_{1}^{\left(r\right)}=\{y_{1}^{\left(r\right)}\left(k\right)=\sum_{i=1}^{k}\frac{(k-i-r-1)!}{(-r-1)!(k-i)!}y_{1}^{\left(0\right)}\left(i\right)|k=1,2,\ldots ,n\}$. *The fractional order accumulation generated sequence of* $Y_{2}^{\left(0\right)}$ *is represented as* $Y_{2}^{\left(r\right)}=\{y_{2}^{\left(r\right)}\left(k\right)=\sum_{i=1}^{k}\frac{(k-i-r-1)!}{(-r-1)!(k-i)!}y_{2}^{\left(0\right)}\left(i\right)|k=1,2,\ldots ,n\}$. *The grey differential equation of FAGM(1,1) can be built by using* $Y_{1}^{\left(0\right)}$ *as follow*:

$$\begin{array}{c} y_{1}^{\left(r\right)}\left(k\right)-y_{1}^{\left(r\right)}\left(k-1\right)+a_{1}z_{1}^{\left(r\right)}\left(k\right)=b_{1}, \end{array}$$ (10)

*where* $z_{1}^{\left(r\right)}\left(k\right)=0.5(y_{1}^{\left(r\right)}\left(k\right)+y_{1}^{\left(r\right)}\left(k-1\right)),k=2,3,\ldots ,n$. *The grey differential equation of FAGM(1,1) can be built by using* $Y_{2}^{\left(0\right)}$ *as follow*:

$$\begin{array}{c} y_{2}^{\left(r\right)}\left(k\right)-y_{2}^{\left(r\right)}\left(k-1\right)+a_{2}z_{2}^{\left(r\right)}\left(k\right)=b_{2}, \end{array}$$ (11)

*where* $z_{2}^{\left(r\right)}\left(k\right)=0.5(y_{2}^{\left(r\right)}\left(k\right)+y_{2}^{\left(r\right)}\left(k-1\right)),k=2,3,\ldots ,n$. *According to the Eqs* (5) *and* (6), *the grey parameters a*_1_ *and b*_1_ *can be obtained as follow*:

$$\begin{array}{c} \begin{array}{cc} a_{1}= & -\frac{\left(n-1\right)\sum_{i=2}^{n}z_{1}^{\left(r\right)}\left(i\right)(y_{1}^{\left(r\right)}\left(i\right)-y_{1}^{\left(r\right)}\left(i-1\right))}{\left(n-1\right)\sum_{i=2}^{n}{\left(z_{1}^{\left(r\right)}\left(i\right)\right)}^{2}-{\left(\sum_{i=2}^{n}z_{1}^{\left(r\right)}\left(i\right)\right)}^{2}}\\ & +\frac{\sum_{i=2}^{n}z_{1}^{\left(r\right)}\left(i\right)\sum_{i=2}^{n}(y_{1}^{\left(r\right)}\left(i\right)-y_{1}^{\left(r\right)}\left(i-1\right))}{\left(n-1\right)\sum_{i=2}^{n}{\left(z_{1}^{\left(r\right)}\left(i\right)\right)}^{2}-{\left(\sum_{i=2}^{n}z_{1}^{\left(r\right)}\left(i\right)\right)}^{2}} \end{array} \end{array}$$ (12)

$$\begin{array}{c} \begin{array}{cc} b_{1}= & -\frac{\sum_{i=2}^{n}z_{1}^{\left(r\right)}\left(i\right)\sum_{i=2}^{n}z_{1}^{\left(r\right)}\left(i\right)(y_{1}^{\left(r\right)}\left(i\right)-y_{1}^{\left(r\right)}\left(i-1\right))}{\left(n-1\right)\sum_{i=2}^{n}{\left(z_{1}^{\left(r\right)}\left(i\right)\right)}^{2}-{\left(\sum_{i=2}^{n}z_{1}^{\left(r\right)}\left(i\right)\right)}^{2}}\\ & +\frac{\sum_{i=2}^{n}{\left(z_{1}^{\left(r\right)}\left(i\right)\right)}^{2}\sum_{i=2}^{n}(y_{1}^{\left(r\right)}\left(i\right)-y_{1}^{\left(r\right)}\left(i-1\right))}{\left(n-1\right)\sum_{i=2}^{n}{\left(z_{1}^{\left(r\right)}\left(i\right)\right)}^{2}-{\left(\sum_{i=2}^{n}z_{1}^{\left(r\right)}\left(i\right)\right)}^{2}} \end{array} \end{array}$$ (13)

*In a similar way, the grey parameters a*_2_ *and b*_2_ *can be obtained as*:

$$\begin{array}{c} \begin{array}{cc} a_{2}= & -\frac{\left(n-1\right)\sum_{i=2}^{n}z_{2}^{\left(r\right)}\left(i\right)(y_{2}^{\left(r\right)}\left(i\right)-y_{2}^{\left(r\right)}\left(i-1\right))}{\left(n-1\right)\sum_{i=2}^{n}{\left(z_{2}^{\left(r\right)}\left(i\right)\right)}^{2}-{\left(\sum_{i=2}^{n}z_{2}^{\left(r\right)}\left(i\right)\right)}^{2}}\\ & +\frac{\sum_{i=2}^{n}z_{2}^{\left(r\right)}\left(i\right)\sum_{i=2}^{n}(y_{2}^{\left(r\right)}\left(i\right)-y_{2}^{\left(r\right)}\left(i-1\right))}{\left(n-1\right)\sum_{i=2}^{n}{\left(z_{2}^{\left(r\right)}\left(i\right)\right)}^{2}-{\left(\sum_{i=2}^{n}z_{2}^{\left(r\right)}\left(i\right)\right)}^{2}} \end{array} \end{array}$$ (14)

$$\begin{array}{c} \begin{array}{cc} b_{2}= & -\frac{\sum_{i=2}^{n}z_{2}^{\left(r\right)}\left(i\right)\sum_{i=2}^{n}z_{2}^{\left(r\right)}\left(i\right)(y_{2}^{\left(r\right)}\left(i\right)-y_{2}^{\left(r\right)}\left(i-1\right))}{\left(n-1\right)\sum_{i=2}^{n}{\left(z_{2}^{\left(r\right)}\left(i\right)\right)}^{2}-{\left(\sum_{i=2}^{n}z_{2}^{\left(r\right)}\left(i\right)\right)}^{2}}\\ & +\frac{\sum_{i=2}^{n}{\left(z_{2}^{\left(r\right)}\left(i\right)\right)}^{2}\sum_{i=2}^{n}(y_{2}^{\left(r\right)}\left(i\right)-y_{2}^{\left(r\right)}\left(i-1\right))}{\left(n-1\right)\sum_{i=2}^{n}{\left(z_{2}^{\left(r\right)}\left(i\right)\right)}^{2}-{\left(\sum_{i=2}^{n}z_{2}^{\left(r\right)}\left(i\right)\right)}^{2}} \end{array} \end{array}$$ (15)

*Based on the relation between the sequence* $Y_{1}^{\left(0\right)}$ *and* $Y_{2}^{\left(0\right)}$, *the equation between the accumulated sequence* $Y_{1}^{\left(r\right)}$ *and* $Y_{2}^{\left(r\right)}$ *are obtained as follow*:

$$\begin{array}{c} y_{2}^{\left(r\right)}\left(k\right)=\sum_{i=1}^{k}\frac{(k-i-r-1)!}{(-r-1)!(k-i)!}y_{1}^{\left(0\right)}\left(i\right)e^{\lambda t}=e^{\lambda t}y_{1}^{\left(r\right)}\left(k\right) \end{array}$$ (16)

*The equation between the sequence* $z_{1}^{\left(r\right)}\left(k\right)$ *and* $z_{2}^{\left(r\right)}\left(k\right)$ *are obtained as follow*:

$$\begin{array}{c} z_{2}^{\left(r\right)}\left(k\right)=0.5(y_{1}^{\left(r\right)}\left(k\right)e^{\lambda t}+y_{1}^{\left(r\right)}\left(k-1\right)e^{\lambda t})=e^{\lambda t}z_{1}^{\left(r\right)}\left(k\right) \end{array}$$ (17)

*Substituting Eqs* (16) *and* (17) *into Eqs* (14) *and* (15), *the equations can be obtained as follow*:

$$\begin{array}{c} \begin{array}{cc} a_{2}= & -\frac{\left(n-1\right)\sum_{i=2}^{n}z_{1}^{\left(r\right)}\left(i\right)e^{\lambda t}(y_{1}^{\left(r\right)}\left(i\right)e^{\lambda t}-y_{1}^{\left(r\right)}\left(i-1\right)e^{\lambda t})}{\left(n-1\right)\sum_{i=2}^{n}{\left(z_{1}^{\left(r\right)}\left(i\right)e^{\lambda t}\right)}^{2}-{\left(\sum_{i=2}^{n}z_{1}^{\left(r\right)}\left(i\right)e^{\lambda t}\right)}^{2}}\\ & +\frac{\sum_{i=2}^{n}z_{1}^{\left(r\right)}\left(i\right)e^{\lambda t}\sum_{i=2}^{n}(y_{1}^{\left(r\right)}\left(i\right)e^{\lambda t}-y_{1}^{\left(r\right)}\left(i-1\right)e^{\lambda t})}{\left(n-1\right)\sum_{i=2}^{n}{\left(z_{1}^{\left(r\right)}\left(i\right)e^{\lambda t}\right)}^{2}-{\left(\sum_{i=2}^{n}z_{1}^{\left(r\right)}\left(i\right)e^{\lambda t}\right)}^{2}}=a_{1} \end{array} \end{array}$$ (18)

$$\begin{array}{c} \begin{array}{cc} b_{2}= & -\frac{\sum_{i=2}^{n}z_{1}^{\left(r\right)}\left(i\right)e^{\lambda t}\sum_{i=2}^{n}z_{1}^{\left(r\right)}\left(i\right)e^{\lambda t}(y_{1}^{\left(r\right)}\left(i\right)e^{\lambda t}-y_{1}^{\left(r\right)}\left(i-1\right)e^{\lambda t})}{\left(n-1\right)\sum_{i=2}^{n}{\left(z_{1}^{\left(r\right)}\left(i\right)e^{\lambda t}\right)}^{2}-{\left(\sum_{i=2}^{n}z_{1}^{\left(r\right)}\left(i\right)e^{\lambda t}\right)}^{2}}\\ & +\frac{\sum_{i=2}^{n}{\left(z_{1}^{\left(r\right)}\left(i\right)e^{\lambda t}\right)}^{2}\sum_{i=2}^{n}(y_{1}^{\left(r\right)}\left(i\right)e^{\lambda t}-y_{1}^{\left(r\right)}\left(i-1\right)e^{\lambda t})}{\left(n-1\right)\sum_{i=2}^{n}{\left(z_{1}^{\left(r\right)}\left(i\right)e^{\lambda t}\right)}^{2}-{\left(\sum_{i=2}^{n}z_{1}^{\left(r\right)}\left(i\right)e^{\lambda t}\right)}^{2}}=b_{1}e^{\lambda t} \end{array} \end{array}$$ (19)

*This proof is completed*.

From Theorem 2, it can be noticed that the grey input varies with time, and the development coefficient remains unchanged if two different subsequences with the same number of samples employed to construct models for a homogeneous exponential sequence. When the grey input linearly changes with time, the typical fractional grey model is the FAGM(1,1,k) model [34] with the linear grey input term *bt* + *c*. Moreover, the input of grey system is non-linear in many other cases. The typical fractional grey model is FTDGM model [51] with nonlinear grey input *t*^*γ*^. Though these models have obtained better prediction performance, the optimization of classical grey models is still required to enhance their adaptability and applicability.

### The power-driven fractional accumulated grey model

Optimization of grey action quantity is an effective and common method to improve the grey model. Theorem 2 shows that the grey input should not be a constant while it should vary with time. Meanwhile, Theorem 2 is derived based on homogeneous exponential time series. In fact, more sequences have the non-homogeneous exponential characteristics in the real world. Therefore, the term *be*^*rt*^ + *c* is considered as the grey action quantity of the proposed fractional accumulated grey model. The definition of the proposed model is represented as follows.

**Definition 3** *The differential equation*

$$\begin{array}{c} \frac{dx^{\left(r\right)}\left(t\right)}{dt}+ax^{\left(r\right)}\left(t\right)=be^{rt}+c. \end{array}$$ (20)

*is the whitening equation of power-driven fractional order accumulated grey model abbreviated as PFAGM, in which a is defined the same in FAGM model, be*^*rt*^ + *c is the power-driven grey action quantity, and r is the fractional order of this grey model*.

Integrating the whitening Eq (20) within [*k* − 1, *k*], we have

$$\begin{array}{c} \int_{k-1}^{k}\frac{dx^{\left(r\right)}\left(t\right)}{dt}dt+a\int_{k-1}^{k}x^{\left(r\right)}\left(t\right)dt=\int_{k-1}^{k}(be^{rt}+c)dt. \end{array}$$ (21)

Then we have

$$\begin{array}{c} x^{\left(r\right)}\left(k\right)-x^{\left(r\right)}\left(k-1\right)+a\int_{k-1}^{k}x^{\left(r\right)}\left(t\right)dt=br^{-1}\left(e^{r}-1\right)e^{r(k-1)}+c. \end{array}$$ (22)

Substituting the background value $z^{\left(r\right)}\left(k\right)=\int_{k-1}^{k}x^{\left(r\right)}\left(t\right)dt$ into Eq (22), the discrete form of PFAGM model can be obtained as follow.

**Definition 4** *The discrete differential equation of PFAGM model is defined as*:

$$\begin{array}{c} x^{\left(r\right)}\left(k\right)-x^{\left(r\right)}\left(k-1\right)+az^{\left(r\right)}\left(k\right)=br^{-1}\left(e^{r}-1\right)e^{r(k-1)}+c. \end{array}$$ (23)

*where z*^(*r*)^ *is the background value, and z*^(*r*)^(*k*) = 0.5*x*^(*r*)^(*k*) + 0.5*x*^(*r*)^(*k* − 1).

When the parameter *b* of PFAGM model is set to 0, PFAGM model can be reduced into FAGM(1,1) model. When the parameter *b* is set to 0 and the fractional order is set to 1, PFAGM model can be degenerated into GM(1,1) model. Because *e*^*γt*^ ≈ 1 + *rt*, PFAGM model can be degenerated into FAGM(1,1,k) model if the term 1 + *rt* replaces the term *e*^*γt*^ of the grey input in PFAGM model.

### Parameter estimation of power-driven factional grey model

To realize the prediction of the fractional grey model, one of the most important problems is parameter estimation for building a model. In fact, it is effective to boost the prediction performance of grey model by using suitable parameters. For PFAGM model, it needs to determine three linear parameters and search out the optimal value of its fractional order. The optimal linear parameters can be gained by using the least-square method under the condition of the given fractional order of PFAGM model. In this subsection, we mainly introduce the principle of linear parameters’ estimation while the methodology of seeking out the optimal fractional order is presented in subsection 3.4.

Once the order of PFAGM model is given, the linear parameters of the PFAGM model can mathematically be calculated as:

$$\begin{array}{c} {\left(a,b,c\right)}^{T}={\left(B_{u}^{T}B_{u}\right)}^{-1}B_{u}^{T}Y_{u}. \end{array}$$ (24)

where

$$\begin{array}{c} Y_{u}=[\begin{array}{c} x^{\left(r\right)}(2)-x^{\left(r\right)}(1)\\ x^{\left(r\right)}(3)-x^{\left(r\right)}(2)\\ \vdots \\ x^{\left(r\right)}(u)-x^{\left(r\right)}(u-1) \end{array}],B_{u}=[\begin{array}{ccc} -z^{\left(r\right)}(2) & r^{-1}(e^{r}-1)e^{r} & 1\\ -z^{\left(r\right)}(3) & r^{-1}(e^{r}-1)e^{2r} & 1\\ \vdots & \vdots & \vdots \\ -z^{\left(r\right)}(u) & r^{-1}(e^{r}-1)e^{(u-1)r} & 1 \end{array}]. \end{array}$$ (25)

in which *u* is the number of samples for fitting. The proof process of parameter estimation is omitted here because it is like that of traditional FAGM model.

### The time response function and restored values

After linear parameters of PFAGM model are calculated, the time response function and the restored values of PFAGM can be got by solving Eq (20). Firstly, we consider the homogeneous differential equation:

$$\begin{array}{c} \frac{dx^{\left(r\right)}\left(t\right)}{dt}+ax^{\left(r\right)}\left(t\right)=0. \end{array}$$ (26)

which is corresponding to the whitening equation of PFAGM model and solve Eq (26). Then the solution of Eq (26) can be obtained as:

$$\begin{array}{c} x^{\left(r\right)}\left(t\right)=Ae^{-at}. \end{array}$$ (27)

where *A* is a constant which is determined by initial condition. Let *x*^(*r*)^(*t*) = *A*(*t*)*e*^−*at*^ be the solution of Eq (20). And substituting it into Eq (20), we have

$$\begin{array}{c} \frac{dA(t)}{dt}e^{-at}=be^{rt}+c. \end{array}$$ (28)

Then the solution of Eq (28) can be easily obtained as:

$$\begin{array}{c} A\left(t\right)=\frac{b}{a+r}e^{(a+r)t}+\frac{c}{a}e^{at}+C. \end{array}$$ (29)

So, the solution of the whitening equation of PFAGM model can be represented as:

$$\begin{array}{c} x^{\left(r\right)}\left(t\right)=Ce^{-at}+\frac{b}{a+r}e^{rt}+\frac{c}{a}. \end{array}$$ (30)

Substituting the initial condition *x*^(*r*)^(1) = *x*^(0)^(1) into Eq (30), the arbitrary constant can be easily determined. And then the time response function of PFAGM model can be derived and represented as:

$$\begin{array}{c} x_{p}^{\left(r\right)}(k)=(x^{\left(0\right)}\left(1\right)-\frac{b}{a+r}e^{r}-\frac{c}{a})e^{-a(k-1)}+\frac{b}{a+r}e^{rk}+\frac{c}{a}. \end{array}$$ (31)

By using inverse *r*-order accumulated operation, the restored value $x_{p}^{\left(0\right)}\left(k\right)$ can be obtained as:

$$x_{p}^{\left(0\right)}(k)={\left(x_{p}^{\left(r\right)}\left(k\right)\right)}^{\left(-r\right)}.$$ (32)

where *k* = 1, 2, …, *n*.

### Searching the optimal order of power-driven fractional accumulated grey model

In subsection 3.2, the linear parameters are estimated by the least-square method under the assumption that the order of fractional power-driven grey model has been given. In fact, the order of PFAGM model needs to be sought out and plays a vital role in boosting its prediction performance effectively. In order to search for an optimal value of the order, we establish a constrained optimization problem of which the objective is minimizing the mean absolute percentage errors for building the grey model. The constraints of PFAGM model have been derived in the above-mentioned modeling process. In summary, the optimization problem can be represented as follow:

$$\begin{array}{c} \begin{array}{c} \min MAPE\left(r\right)=\frac{1}{u-1}\sum_{i=2}^{u}|\frac{{x_{p}}^{\left(0\right)}\left(i\right)-x^{\left(0\right)}\left(i\right)}{x^{\left(0\right)}\left(i\right)}|\times 100\%.\\ s.t.\{\begin{array}{c} {\left(a,b,c\right)}^{T}={\left(B_{u}^{T}B_{u}\right)}^{-1}B_{u}^{T}Y_{u}.\\ x_{p}^{\left(r\right)}(k)=(x^{\left(0\right)}\left(1\right)-\frac{b}{a+r}e^{r}-\frac{c}{a})e^{-a(k-1)}+\frac{b}{a+r}e^{rk}+\frac{c}{a}.\\ x^{\left(0\right)}\left(k\right)={\left(x_{p}^{\left(r\right)}\right)}^{\left(-r\right)},k=2,3,\ldots ,u. \end{array} \end{array} \end{array}$$ (33)

where *B*_*u*_ and *Y*_*u*_ are defined as Eq (25). It can be clearly noticed that the optimization problem Eq (33) is a complex nonlinear programming problem with nonlinear objective function and a few nonlinear constraints. Obviously, it is difficult to derive the exact solution for optimal fractional order. However, the optimal value of fractional order plays a very important roles in boosting the prediction accuracy of existing grey models dramatically. For example, Ma et al. established a similar optimization problem to optimize the fractional order of FTDGM model and applied it to forecast energy consumption accurately [51]. Wu et al. also build a similar optimization problem to obtain the optimal fractional order and power index of FANGBM and forecasted the renewable energy consumption successfully [37]. These facts have shown that the optimal fractional order can be used to boost the prediction performance of grey models.

In order to obtain the optimal order of PFAGM, we adopted a nature-based heuristic intelligent method called Whale Optimization Algorithm (WOA) to solve the nonlinear programming problem Eq (33). WOA algorithm was firstly proposed by Mirjalili et al. in 2016 [56]. The inspiration of WOA is from the social behaviors of whales. It mimics the bubble-net feeding strategy including shrinking encircling, spiral updating position and randomly hunting behavior. Due to the simple rules and local optimization performance of WOA, it is widely used in many domains such as feature selection, clustering, classification, image processing and so on [57]. In this paper, it is assumed by WOA that there is a population with 30 humpback whales as search agents in search space. *P*(*k*) indicates the position vector of search agents at iteration *k*. P*(*k*) represents the candidate solution which is the best one or near to the optimum. The procedures of searching optimal fractional order with WOA are depicted as follow:

**Step 1**: Initialize the initial position *P*(*k*) of search agent randomly in search space at *k* = 1 in search space. And set maximum iterations *T*_*m*_ to be 300. The value of each agent’s position indicates a possible fractional order of PFAGM model.
**Step 2**: Compute the fitness of each agent and obtain the candidate solution P*(*k*) at *k* = 1. According to Eq 33, the fitness function is defined as:
  $$\begin{array}{c} Fitness=\frac{1}{u-1}\sum_{i=2}^{u}|\frac{x_{p}^{\left(0\right)}\left(i\right)-x^{\left(0\right)}\left(i\right)}{x^{\left(0\right)}\left(i\right)}|\times 100\%. \end{array}$$ (34)
  The position of search agent with minimum fitness is considered as the candidate solution at first iteration.
**Step 3**: Update the position vectors of humpback whales. When humpback whales forage the prey, they usually move around the prey by shrinking encircling and spiral updating position simultaneously. In order to imitate this simultaneous behavior, we assume that there is a 50% probability to shrink encircling or update position spirally in the iteration of optimization. In each iteration, it generates a random number in [0, 1] and set it to the parameter *p*. If *p* ≤ 0.5, humpback whales select to shrink encircling. Mathematically, the behavior of shrinking encircling can be represented as follows:
  $$\vec{A}=2g\left(k\right)\cdot \vec{r}-g\left(k\right).$$ (35)
  $$\vec{B}=\left|2\cdot \vec{r}\cdot {\vec{P}}^{*}\left(k\right)-\vec{P}\left(k\right)\right|.$$ (36)
  $$\vec{P}\left(k+1\right)={\vec{P}}^{*}\left(k\right)-\vec{A}\cdot \vec{B}.$$ (37)
  $$\begin{array}{c} g\left(k\right)=2-2k/T_{m}. \end{array}$$ (38)
  where $\vec{r}$ is a random vector in interval [0, 1]. If *p* > 0.5, the humpback whales will select spiral updating position to imitate the helix-shaped moving behavior in process of optimization. The behavior is shown mathematically as follow:
  $$\vec{P}\left(k+1\right)=\left|{\vec{P}}^{*}\left(k\right)-\vec{P}\left(k\right)\right|\cdot e^{\omega l}\cdot \text{cos}\left(2\pi l\right)+{\vec{P}}^{*}\left(k\right).$$ (39)
  where *l* denotes a random value in [−1, 1], *ω* is a constant. In fact, the humpback whales search for the prey randomly. If $|\vec{A}|>1$, WOA algorithm imitates the behavior and performs a global search. Mathematically, the model is formulated as follows:
  $$\vec{C}=\left|2\cdot r\cdot {\vec{P}}_{rand}\left(k\right)-\vec{P}\left(k\right)\right|.$$ (40)
  $$\vec{P}\left(k+1\right)={\vec{P}}_{rand}\left(k\right)-\vec{A}\cdot \vec{C}.$$ (41)
  where ${\vec{P}}_{rand}(k)$ is the position of a random search agent.
**Step 4**: Calculate the fitness of each humpback whales. If there is a better candidate with minimum fitness value, it is needed to update the optimal solution ${\vec{P}}^{*}$.
**Step 5**: If the ending condition is satisfied, the optimal fractional order is obtained, otherwise *k* = *k* + 1.

**Fig 1:**
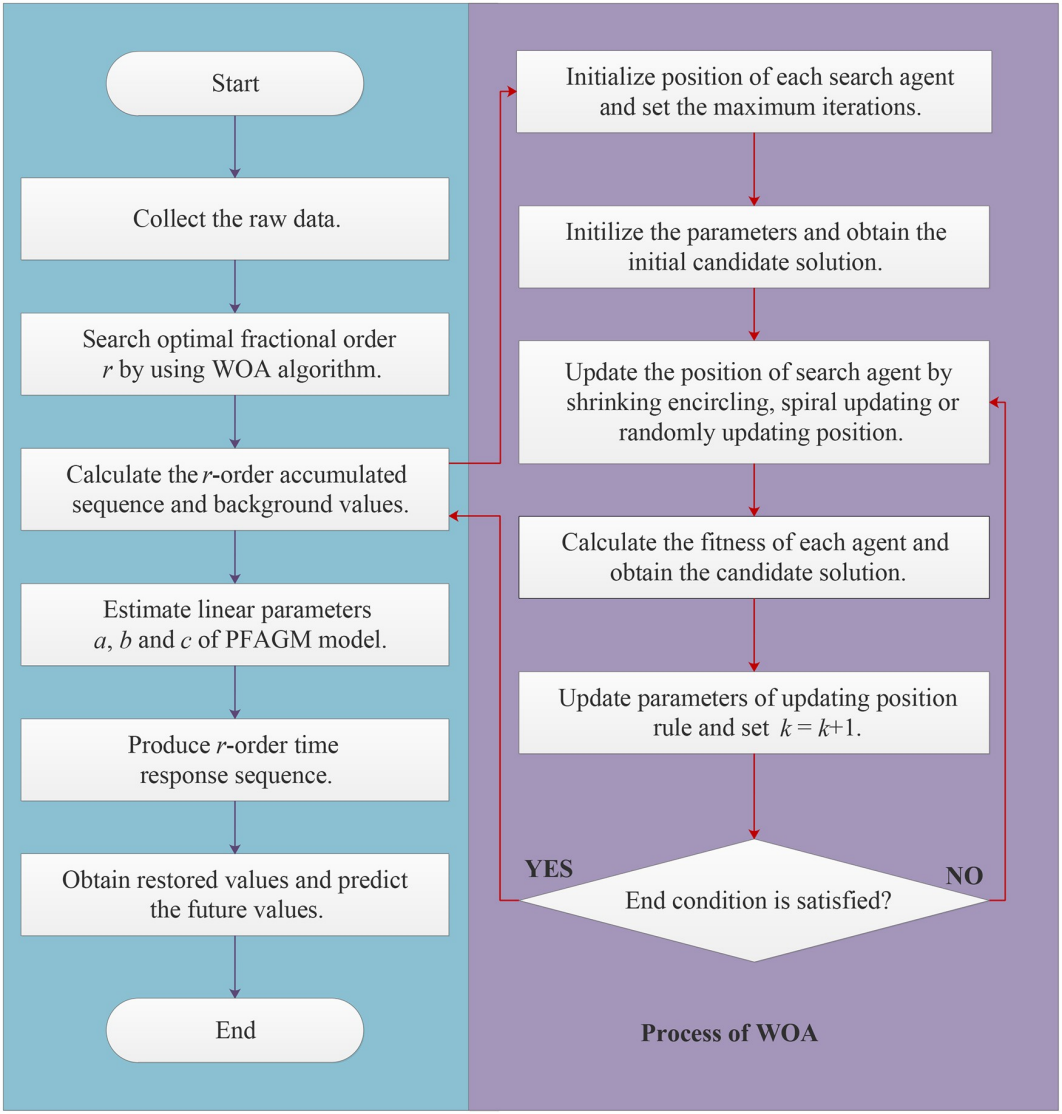
The flowchart of PFAGM model.

Based on the process of building PFAGM model and optimization of the fractional order, the detailed modeling procedure of power-driven fractional accumulated grey model can be shown in Fig 1. For enhancing the prediction performance of PFAGM model, the key is to obtain the optimal values of linear parameters and fractional order. The optimal value of fractional order can be sought out through solving the established optimization problem by WOA algorithm. Moreover, the linear parameters are estimated by using the least-square method. Then the established PFAGM model is used to predict future values in different applications.

### Applicability analysis of power-driven fractional grey model

Though the classical grey models are effective approaches to predict future values with small samples, each of these models has a certain scope of application. Similarly, the proposed power-driven fractional grey model also has its scope of application. Therefore, we construct a series of sequences, including two kinds of non-homogeneous exponential sequences and some other sequences with special shapes, to study the applicability of the proposed grey model. There are three cases studied and compared with GM(1,1), FAGM(1,1), and FAGM(1,1,k) as follows.

Firstly, we construct a series of non-homogeneous exponential sequences with the larger values of the development coefficient. These sequences are represented as *x*^(0)^(*k*) = *Ae*^λ*t*^ + *B*(*k* = 1, 2, …, 9) in which *A* is set to 1, *B* is set to 10, and *a* is set to 0.7, 1.0, 1.3, 1.5, 1.7 or 1.8. The first six digits of each sequence are used to train the grey models. The other three digits of each sequence are employed to examine the prediction performance of the proposed grey model. The MAPEs of different grey models for fitting and prediction are filled in Table 1. It can be noticed that PFAGM model obtains a significant advantage of prediction accuracy over the other three classical grey models. Meanwhile, PFAGM model has significant advantages in fitting. Secondly, we construct the other kind of non-homogeneous exponential sequences represented as *x*^(0)^(*k*) = *Ae*^λ*t*^ + *B*(*k* − 1)(*k* = 1, 2, …, 9) in which *A* is set to 1, *B* is set to 5, and *a* is set to 0.7, 1.0, 1.3, 1.5, 1.7 or 1.8. Similarly, the first six digits of each sequence are used to train the grey models. The other three digits of each sequence are employed to examine the prediction performance of the proposed grey model. Table 2 shows the MAPEs of different grey models for fitting and prediction. The results indicate that PFAGM model has a significant advantage of fitting ability over the other three classical grey models. PFAGM model also has a significant superiority of prediction accuracy, especially when the development coefficient is large. From the above analysis results, it indicates that PFAGM model has better adaptability and applicability for the two kinds of non-homogeneous exponential sequences with the larger values of the development coefficient.

**Table 1:** Comparison of different grey models for non-homogeneous exponential sequences with various development coefficients (*X*^(0)^(*k*) = *Ae*^λ(*k*−1)^ + *B*, *k* = 1, 2, ⋅, *n*).

λ	MAPE of Fitting				MAPE of Prediction			
	GM(1,1)	FAGM(1,1)	FAGM(1,1,k)	PFAGM	GM(1,1)	FAGM(1,1)	FAGM(1,1,k)	PFAGM
**0.7**	8.0214	1.4179	2.0788	**0.3766**	43.4858	5.1797	26.5822	**3.9745**
**1**	69.4463	4.6622	5.2342	**3.38E-07**	88.7597	19.0313	27.3230	**1.61E-06**
**1.3**	188.7666	10.4601	11.3918	**1.0518**	180.0536	46.3293	45.6655	**0.9277**
**1.5**	237.1642	15.7192	17.0864	**1.9898**	193.0147	59.6113	59.0422	**2.3112**
**1.7**	249.5205	22.4229	24.1564	**3.0308**	171.0404	71.1620	70.8141	**3.9040**
**1.8**	246.3506	26.4090	28.2787	**3.5722**	156.4693	76.0759	75.8225	**4.7317**

**Table 2:** Comparison of different grey models for non-homogeneous exponential sequences with various development coefficients (*X*^(0)^(*k*) = *Ae*^λ(*k*−1)^ + *B*(*k* − 1), *k* = 1, 2, ⋅, *n*).

λ	MAPE of Fitting				MAPE of Prediction			
	GM(1,1)	FAGM(1,1)	FAGM(1,1,k)	PFAGM	GM(1,1)	FAGM(1,1)	FAGM(1,1,k)	PFAGM
**0.7**	5.4555	2.7347	2.4818	**0.8435**	14.2598	22.5767	14.4327	**4.6936**
**1**	26.9863	13.4895	9.2376	**2.4296**	54.5762	44.7105	36.2111	**23.0307**
**1.3**	111.0393	10.5668	9.9856	**3.6362**	119.3470	29.8071	29.0046	**15.3936**
**1.5**	163.5235	9.7733	9.6019	**3.9296**	144.4912	45.4235	45.0679	**9.4585**
**1.7**	190.9126	13.5743	13.7557	**3.8576**	142.1207	58.5609	58.0279	**4.6953**
**1.8**	195.8534	16.2803	16.0250	**3.7240**	135.6079	72.4797	72.4032	**3.0292**

**Fig 2:**
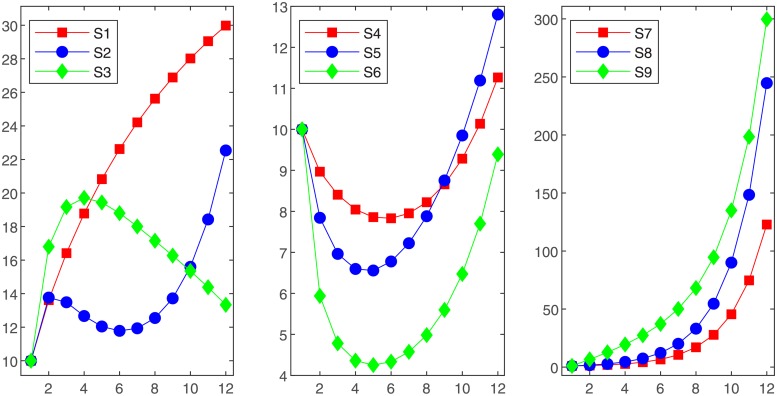
Different non-homogeneous exponential sequences.

Finally, we generate a series of non-homogeneous exponential sequences with different characteristics randomly. The raw data of each sequence is tabulated in Table 3. Fig 2 exhibits the characteristics of these generated sequences. These sequences are divided into two groups. The first group, including the first eight digits of each sequence, is used to build grey models. The second group, including the other four digits of each sequence, is utilized to validate the prediction performance of the proposed grey model. The MAPEs of the grey models for fitting and prediction are filled in Table 4. It can be noticed that the prediction accuracy of the proposed grey model is better than those of the other three grey models. The fitting errors of the proposed grey model are the lowest or very approximate to the lowest.

**Table 3:** Raw data of special non-homogeneous exponential sequences.

Index	S1	S2	S3	S4	S5	S6	S7	S8	S9
**1**	10	10	10	10	10	10	1	1	1
**2**	13.6138	13.7664	16.7975	8.9671	7.8421	5.9371	1.32435	1.6487	6.6487
**3**	16.4143	13.4857	19.1673	8.4053	6.9623	4.7812	1.85915	2.7183	12.7183
**4**	18.7775	12.6666	19.7109	8.0436	6.5948	4.359	2.74085	4.4817	19.4817
**5**	20.8251	12.0434	19.4351	7.8582	6.5571	4.2515	4.19455	7.3891	27.3891
**6**	22.6213	11.7834	18.7967	7.8306	6.7771	4.3335	6.59125	12.1825	37.1825
**7**	24.2093	11.9347	18.0058	7.9513	7.2224	4.5737	10.54275	20.0855	50.0855
**8**	25.6219	12.5487	17.153	8.2224	7.8808	4.9824	17.05775	33.1155	68.1155
**9**	26.8853	13.7188	16.2686	8.658	8.7532	5.5959	27.7991	54.5982	94.5982
**10**	28.0212	15.5993	15.3514	9.2834	9.85	6.4732	45.50855	90.0171	135.0171
**11**	29.0477	18.4248	14.3833	10.1355	11.19	7.6988	74.7066	148.4132	198.4132
**12**	29.9801	22.5364	13.3348	11.2646	12.7997	9.3895	122.846	244.6919	299.6919

**Table 4:** Comparison of different grey models for special non-homogeneous exponential sequences.

a	MAPE of Fitting				MAPE of Prediction			
	GM(1,1)	FAGM(1,1)	FAGM(1,1,k)	PFAGM	GM(1,1)	FAGM(1,1)	FAGM(1,1,k)	PFAGM
**S1**	3.1505	0.0471	0.0311	0.0022	18.4623	0.2208	0.2560	**0.0221**
**S2**	2.7926	2.0018	1.4507	0.4060	34.0582	30.6134	25.5486	**6.6880**
**S3**	4.4282	0.2048	0.1991	0.1987	22.2005	2.2671	1.0236	**0.5911**
**S4**	2.5212	0.4465	0.0444	0.0236	22.5855	28.3267	1.9441	**1.2779**
**S5**	5.2952	0.6137	0.0218	0.1080	30.1755	16.2743	1.7891	**0.6995**
**S6**	7.7229	0.5409	0.1851	0.2357	41.5405	3.4184	10.1779	**1.8713**
**S7**	15.0169	1.5770	2.2206	0.5789	25.0665	5.1843	8.6351	**2.3063**
**S8**	4.7913	1.8546	2.3858	0.2575	10.6270	5.3084	7.8210	**0.7552**
**S9**	9.0649	2.1075	1.0262	0.5335	5.8343	19.3921	6.9563	**4.4094**

From the above validations and analysis, it can be drawn that PFAGM model has a significant superiority to deal with the non-homogeneous exponential sequences (formulated as *X*^(0)^(*k*) = *Ae*^λ(*k*−1)^ + *B*) or *X*^(0)^(*k*) = *Ae*^λ(*k*−1)^ + *B*(*k* − 1)) compared with the classical GM(1,1), FAGM(1,1) and FAGM(1,1,k) model. Meanwhile, PFAGM model has a certain advantage to handle the above-mentioned special sequences. Therefore, PFAGM model has better adaptability and applicability than the other classical grey models for non-homogeneous exponential sequences with larger development coefficients or special shape characteristics.

## Validation of the power-driven grey model

### Performance metrics and comparative grey models

In order to evaluate the accuracy of grey prediction models, four performance metrics are adopted in numerical validation and case studies, including residual percentage error (RPE), absolute percentage error (APE), mean absolute percentage error (MAPE) and Correlation coefficient (R) [58, 59]. The metrics RPE and APE are used to validate the prediction accuracy of a grey model for a single data point. Mathematically, they are represented as follows:

$$\begin{array}{c} RPE\left(k\right)=\frac{x_{p}^{\left(0\right)}(k)-x^{\left(0\right)}(k)}{x^{\left(0\right)}(k)}\times 100\%. \end{array}$$ (42)

$$\begin{array}{c} APE\left(k\right)=|RPE(k)|. \end{array}$$ (43)

where *x*^(0)^(*k*) is raw data, $x_{p}^{\left(0\right)}(k)$ is the value produced by a grey model. Meanwhile, MAPE is used as a general metric to evaluate the accuracy of a prediction model. The lower value of MAPE indicates that the model has a better performance. Mathematically, MAPE is represented as follow:

$$\begin{array}{c} MAPE=\frac{1}{n}\sum_{k=1}^{n}|APE(k)|. \end{array}$$ (44)

where *n* is the number of samples. For a raw sequence with n samples, the metric FMAPE is defined as the simulation performance metric while PMAPE is defined as a prediction performance metric. Mathematically, they can be formulated as:

$$\begin{array}{c} FMAPE=\frac{1}{v}\sum_{k=1}^{v}|APE(k)|. \end{array}$$ (45)

$$\begin{array}{c} PMAPE=\frac{1}{n-v}\sum_{k=v+1}^{n}|APE(k)|. \end{array}$$ (46)

where *v* is the number of samples used to build a model while the rest of the raw sequence is used to examine the prediction accuracy of the model. The total MAPE is used to evaluate the whole performance of a model and abbreviated as TMAPE. The correlation coefficient (R) is used to describe the relationship between raw sequence *X*^(0)^ and the sequence $X_{p}^{\left(0\right)}$ produced by grey models. The mathematical definition of R is represented as:

$$\begin{array}{c} R=\frac{Cov(X^{\left(0\right)},X_{p}^{\left(0\right)})}{\sqrt{Var\left(X^{\left(0\right)}\right)Var\left(X_{p}^{\left(0\right)}\right)}}. \end{array}$$ (47)

where *Cov*(*s*, *t*) denotes the covariance of sequence *s* and *t*, *Var*(*s*) denotes the variances of sequence *s*.

For investigating its superiority of performance, PFAGM model compares with three existing grey models, including traditional integral-order grey model (GM(1, 1)), fractional accumulated grey model (FAGM(1,1)) and the improved fractional accumulated grey model (FAGM(1,1,k)). The whitening equation of integral-order GM(1,1) model [38] is represented as:

$$\begin{array}{c} \frac{dx^{\left(1\right)}\left(t\right)}{dt}+ax^{\left(1\right)}\left(t\right)=b. \end{array}$$ (48)

The whitening equation of FAGM(1,1,k) [34] is written as:

$$\begin{array}{c} \frac{dx^{\left(r\right)}\left(t\right)}{dt}+ax^{\left(r\right)}\left(t\right)=bt+c. \end{array}$$ (49)

The details of FAGM(1,1) model are introduced in section 2. All grey prediction models, including the new proposed model and the three contrast grey models, are implemented in MATLAB and performed on the platform MATLAB 2018. In particular, it needs to highlight that the orders of the two comparative fractional grey models are also sought out by the optimization algorithm WOA which also is applied in our proposed PFAGM model. In the following subsections, the validation experiments on some real-world data sets are conducted to illustrate the advantages of PFAGM compared with the other three existing grey models.

### Example A: Forecasting China’s nuclear energy consumption

**Fig 3:**
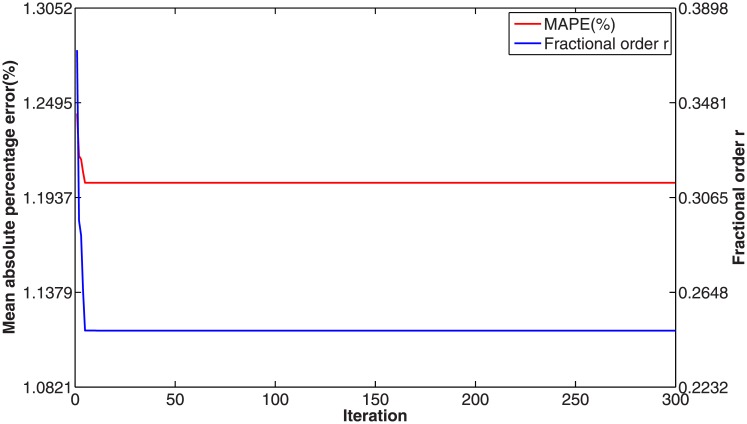
Searching optimal fractional order of PFAGM by using WOA for Example A.

**Fig 4:**
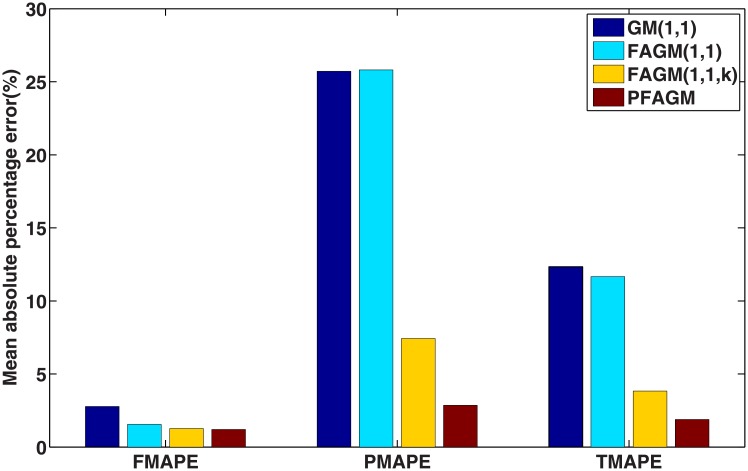
Performance comparison of the proposed model and other comparative grey models in Example A.

**Fig 5:**
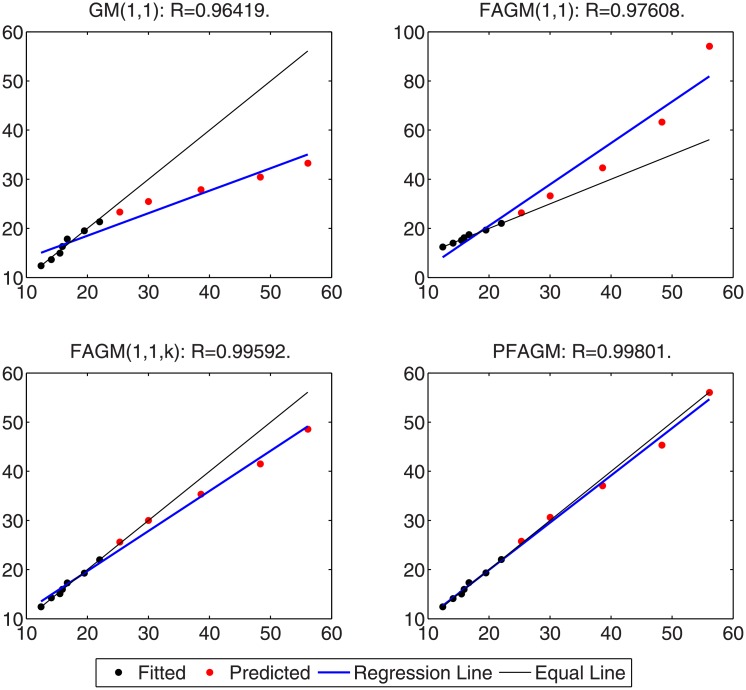
Analysis of detailed results obtained by using the proposed model and other comparative grey models in Example A.

In this numerical validation, the raw sequence containing Chinese nuclear energy consumption (NEC) from 2006 to 2017 is obtained from section 8 of reference [34]. The dataset is tabulated in Table 5. The consumption from 2006 to 2012 is used to build a model respectively for PFAGM model and the other three contrast grey models, while the remainder of the dataset is used to validate the prediction performance of grey models. Firstly, we obtain the optimal orders of the three fractional grey models by using WOA algorithm. Then the linear parameters of all grey models are estimated by using the least-square method. The optimal values of linear and nonlinear parameters for each model are filled in Table 6. From Fig 3, it can be noticed that WOA algorithm converges rapidly into a stable status after a few iterations. And the optimal order of PFAGM model is obtained and equal to 0.24794. The results produced by these established grey models are also filled in Table 7. FMAPE, PMAPE, and TMAPE of PFAGM model are respectively 1.2024, 2.8591, and 1.8927. As can be noticed from Fig 4 and Table 7, all evaluation metrics’ values of PFAGM model are lowest compared with those of the other three contrast grey models. Fig 5 shows the detailed analysis between the raw data and the values produced by the four grey models. It can be clearly found that the linear regression line obtained from PFAGM model almost coincides with the equal line of which the point denotes that raw data is equal to the value produced by the grey model. It indicates that PFAGM model has a better prediction performance than the other three contrast models.

**Table 5:** Raw data in Example A.

Year	NEC	Year	NEC	Year	NEC
2006	12.4	2010	16.7	2014	30
2007	14.1	2011	19.5	2015	38.6
2008	15.5	2012	22	2016	48.3
2009	15.9	2013	25.3	2017	56.1

**Table 6:** The optimal parameters of different grey models in Example A.

Parameters	GM(1,1)	FAGM(1,1)	FAGM(1,1,k)	PFAGM
*a*	-0.08907	-0.50681	0.07111	0.80372
*b*	11.94888	-6.15082	0.67748	3.86999
*c*	-	-	1.99915	11.19314
*γ*	1	-0.11349	-0.2949	0.24794

**Table 7:** The results produced by proposed model and other comparative grey models in Example A.

Year	Raw data	GM(1,1)		FAGM(1,1)		FAGM(1,1,k)		PFAGM	
		Value	RPE	Value	RPE	Value	RPE	Value	RPE
2006	12.4	12.4	0	12.4	0	12.4	0	12.4	0
2007	14.1	13.6523	3.1752	13.9812	0.8422	14.2607	-1.1397	14.1	0
2008	15.5	14.9241	3.7156	15.0733	2.7527	15.0798	2.7112	15.0037	3.2022
2009	15.9	16.3143	-2.6059	16.1486	-1.5633	15.9525	-0.3303	15.9486	-0.3059
2010	16.7	17.8341	-6.7911	17.4546	-4.5184	17.2798	-3.4716	17.3112	-3.6598
2011	19.5	19.4955	0.0233	19.2689	1.1854	19.259	1.2359	19.2864	1.0955
2012	22	21.3116	3.1293	22	0	22	0	22.0337	-0.1531
2013	25.3	23.2969	7.9176	26.3009	-3.9563	25.5662	-1.0521	25.736	-1.7235
2014	30	25.4671	15.1097	33.2414	-10.8046	29.9935	0.0215	30.6283	-2.0943
2015	38.6	27.8395	27.877	44.5882	-15.5136	35.3004	8.5482	37.0175	4.0998
2016	48.3	30.4329	36.992	63.269	-30.9917	41.4932	14.0928	45.3032	6.2045
2017	56.1	33.2679	40.699	94.1392	-67.806	48.5701	13.4223	56.0027	0.1734
FMAPE		2.7772		1.5517		1.2698		1.2024	
PMAPE		25.7191		25.8144		7.4274		**2.8591**	
TMAPE		12.3363		11.6612		3.8355		**1.8927**	
R		0.9642		0.9761		0.9959		**0.998**	

### Example B: Forecasting cumulative oil field production

**Fig 6:**
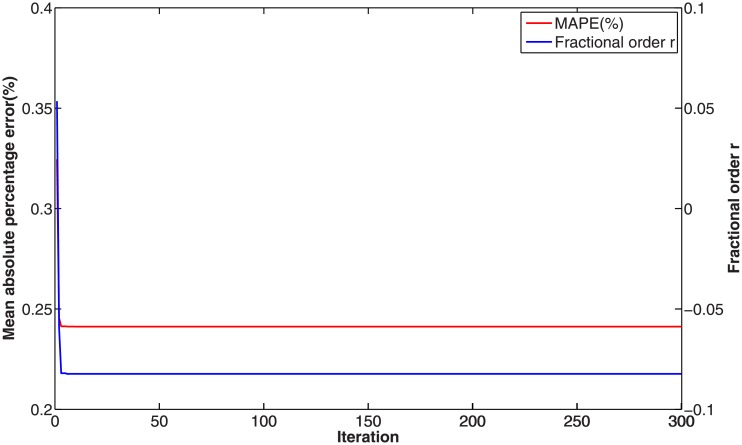
Searching optimal fractional order of PFAGM by using WOA for Example B.

**Fig 7:**
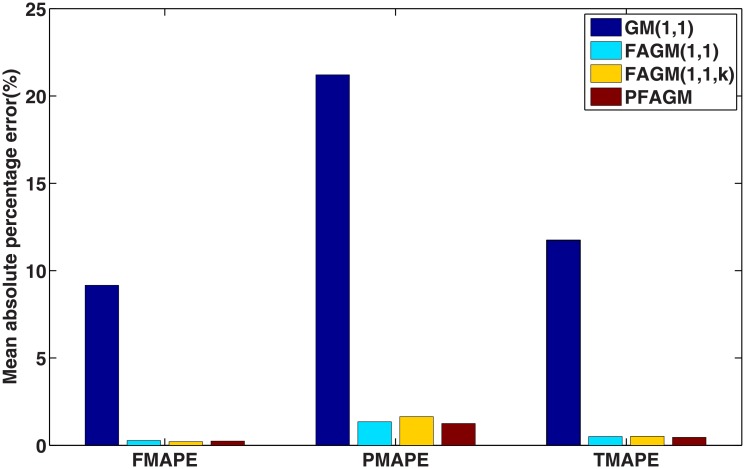
Performance comparison of the proposed model and other comparative grey models in Example B.

**Fig 8:**
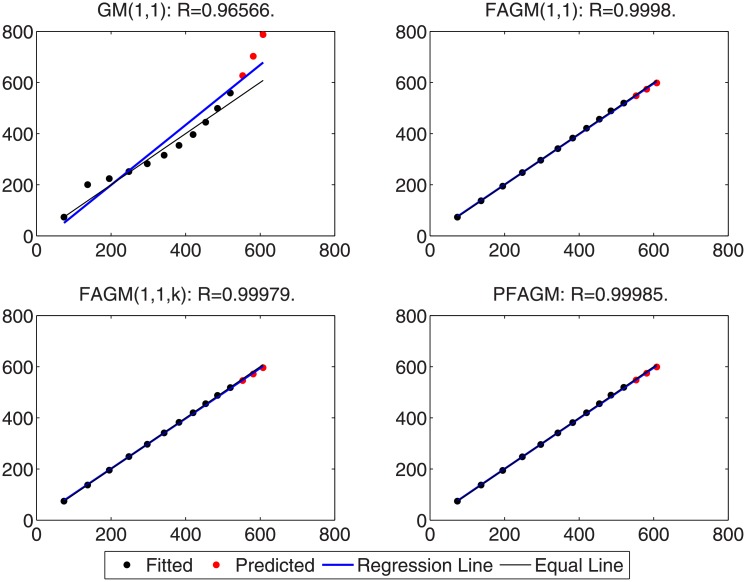
Analysis of detailed results obtained by using the proposed model and other comparative grey models in Example B.

In this subsection, we consider forecasting the cumulative oil field production of which the raw data is collected from the paper [60] as a validation example. The raw data contains the cumulative oil field production from 1999 to 2012 of the RQ block of Huabei oil field company in China. The raw data is tabulated in Table 8. Then the dataset is divided into two subsets. The first subset, including the first 11 samples, is used to build a model for each grey model. The other subset, including the last 3 samples, is utilized to examine the accuracy of grey models. In the stage of building model, the optimal orders of all fractional grey models are obtained by using WOA algorithm. From Fig 6, it can be obviously noticed that the optimal order of PFAGM is sought out after a few iterations of WOA. And then the linear parameters of these models are also obtained by using the least-square method after their orders are determined. The parameters of the integral order grey model can be directly estimated by using the least-square method. These optimal parameters are filled in Table 9. Then we utilize these different established grey models to calculate the fitted production from 1999 to 2009 and the predicted production from 2010 to 2012, which are filled in Table 10. As can be noticed from Table 10 and Fig 7, the proposed PFAGM model exhibits the most excellent prediction performance compared with those of the other contrast grey models. Though FMAPE of PFAGM model is not best, its PMAPE and TMAPE are superior to the other three contrast models. R of FMAPE is also best among those of these grey models. Meanwhile, it is shown that results produced by PFAGM almost approximate to the real value, and the regression line is almost coincident with the equal line for raw sequence and the produced sequence by the model in Fig 8. Above all, PFAGM model is slightly superior to the other three grey models in the aspect of prediction accuracy.

**Table 8:** Raw data of cumulative oil field production in example B.

Year	Oil production	Year	Oil production	Year	Oil production
1999	73.8217	2004	342.6394	2009	519.8508
2000	136.8817	2005	382.4312	2010	552.6569
2001	195.059	2006	420.0399	2011	581.6092
2002	247.8547	2007	454.043	2012	608.1863
2003	297.0902	2008	485.1171		

**Table 9:** The optimal parameters of different grey models in Example B.

Parameters	GM(1,1)	FAGM(1,1)	FAGM(1,1,k)	PFAGM
*a*	-0.1142	0.0689	0.0513	0.3618
*b*	180.4262	73.4323	81.0772	-172.2099
*c*	-	-	31.5459	246.4802
*γ*	1	0.04046	1.12273	-0.08235

**Table 10:** The results produced by proposed model and other comparative grey models in Example B.

Year	Raw data	GM(1,1)		FAGM(1,1)		FAGM(1,1,k)		PFAGM	
		Value	RPE	Value	RPE	Value	RPE	Value	RPE
1999	73.8217	73.8217	0	73.8217	0	73.8217	0	73.8217	0
2000	136.8817	200.0595	46.155	136.8817	0	136.8817	0	136.8817	0
2001	195.059	224.2578	14.9692	194.4272	-0.3239	195.7859	0.3727	194.6208	-0.2247
2002	247.8547	251.3831	1.4236	247.2619	-0.2392	248.754	0.3628	247.4707	-0.1549
2003	297.0902	281.7893	-5.1502	295.9235	-0.3927	297.0427	-0.016	295.9658	-0.3785
2004	342.6394	315.8733	-7.8117	340.8246	-0.5296	341.3715	-0.37	340.6058	-0.5935
2005	382.4312	354.08	-7.4134	382.305	-0.033	382.2394	-0.0501	381.8222	-0.1592
2006	420.0399	396.908	-5.5071	420.656	0.1467	420.025	-0.0036	419.9784	-0.0146
2007	454.043	444.9163	-2.0101	456.1335	0.4604	455.0314	0.2177	455.3797	0.2944
2008	485.1171	498.7315	2.8064	488.9656	0.7933	487.5107	0.4934	488.2846	0.6529
2009	519.8508	559.0559	7.5416	519.3578	-0.0948	517.6779	-0.418	518.9141	-0.1802
2010	552.6569	626.6769	13.3935	547.4965	-0.9337	545.7198	-1.2552	547.4598	-0.9404
2011	581.6092	702.477	20.7816	573.5516	-1.3854	571.8011	-1.6864	574.0897	-1.2929
2012	608.1863	787.4456	29.4744	597.6785	-1.7277	596.0686	-1.9924	598.9529	-1.5182
FMAPE		9.1626		0.274		**0.2095**		0.2412	
PMAPE		21.2165		1.349		1.6447		**1.2505**	
TMAPE		11.7456		0.5043		0.517		**0.4575**	
R		0.9657		0.9998		0.9998		**0.9999**	

### Example C: Forecasting foundation settlement

**Fig 9:**
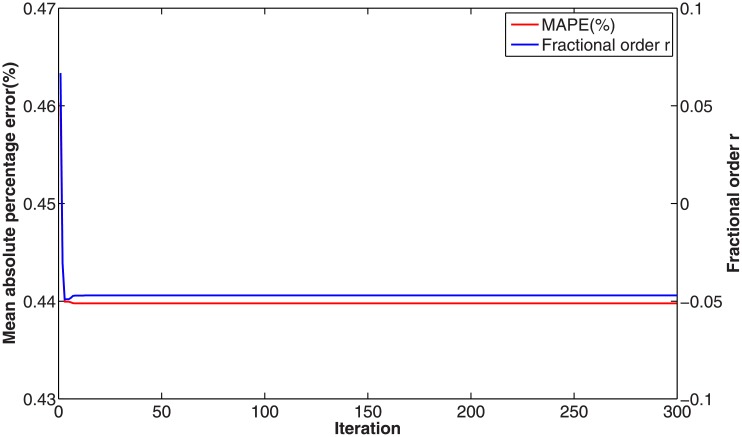
Searching optimal fractional order of PFAGM by using WOA for Example C.

**Fig 10:**
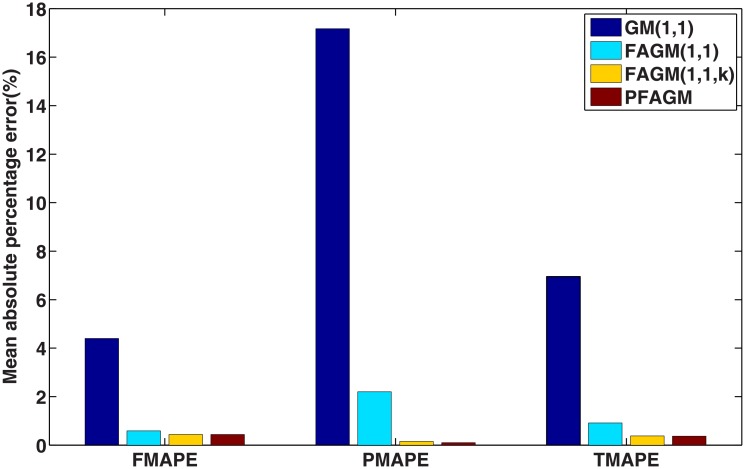
Performance comparison of the proposed model and other comparative grey models in Example C.

**Fig 11:**
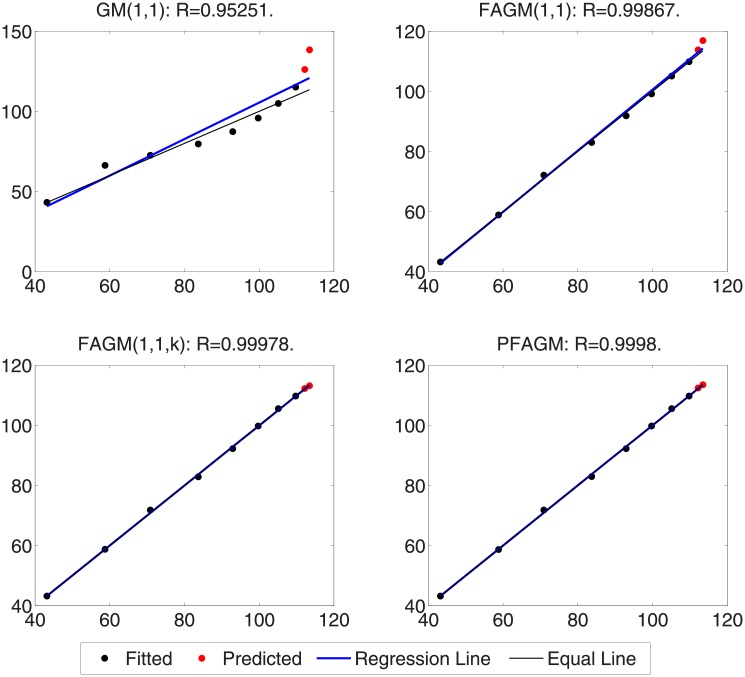
Analysis of detailed results obtained by using the proposed model and other comparative grey models in Example C.

In this validation, the raw data of foundation settlement during engineering construction is obtained from the paper [61], which is tabulated in Table 11. According to the previous practices, we divide the raw into two groups. The first one consists of the first eight digits of foundation settlement, which are used to build grey models for the proposed model and the contrast grey models. The second one consists of the last two digits used to validate the prediction accuracy of each grey model. Firstly, the optimal orders of the fractional accumulated grey models are found out by using WOA algorithm. Then their linear parameters are estimated by using the least-square method under their determined fractional order. The parameters of GM(1,1) model are obtained by the least-square method directly. These optimal parameters are listed in Table 12. In Fig 9, it is shown that the convergence curve of WOA declines rapidly and then stays a constant stably after about 20 iterations. The results produced by these grey models are also tabulated in Table 13. From Table 13 and Fig 10, it can be apparently found that PFAGM model has the lowest PMAPE and TMAPE compared with the other three contrast grey models and has the second-lowest FMAPE which is almost approximate to the lowest FMAPE of FAGM(1,1,k) model. Meanwhile, it can be clearly seen from Fig 11 that the regression line between raw data and calculated values is almost coincident with their equal line. In brief, it can be concluded that PFAGM model has better prediction performance than the other three grey models in this example.

**Table 11:** Raw data of foundation settlement in Example C.

Index	Observed value	Index	Observed value	Index	Observed value
1	43.19	6	99.73	9	112.19
2	58.73	7	105.08	10	113.45
3	70.87	8	109.73		
4	83.71	5	92.91		

**Table 12:** The optimal parameters of different grey models in Example C.

Parameters	GM(1,1)	FAGM(1,1)	FAGM(1,1,k)	PFAGM
*a*	-0.0920	0.1274	0.0421	0.0126
*b*	59.2681	30.1302	-1.3201	42.9745
*c*	-	-	15.6763	-25.9805
*γ*	0.95251	0.99867	0.99978	0.99980

**Table 13:** The results produced by proposed model and other comparative grey models in Example C.

Index	Raw data	GM(1,1)		FAGM(1,1)		FAGM(1,1,k)		PFAGM	
		Value	RPE	Value	RPE	Value	RPE	Value	RPE
1	43.19	43.19	0	43.19	0	43.19	0	43.19	0
2	58.73	66.243	12.7925	58.8407	0.1884	58.73	0	58.6616	-0.1164
3	70.87	72.6275	2.4798	72.1069	1.7453	71.7718	1.2724	71.8027	1.3161
4	83.71	79.6272	-4.8773	82.9702	-0.8838	82.8814	-0.9899	82.9569	-0.8997
5	92.91	87.3016	-6.0364	91.8531	-1.1376	92.1827	-0.7828	92.2435	-0.7173
6	99.73	95.7156	-4.0253	99.1141	-0.6176	99.7299	-0.0001	99.7491	0.0191
7	105.08	104.9405	-0.1327	105.0387	-0.0393	105.5627	0.4593	105.5524	0.4496
8	109.73	115.0546	4.8524	109.8553	0.1141	109.7181	-0.0108	109.73	0
9	112.19	126.1434	12.4373	113.749	1.3896	112.2341	0.0393	112.3567	0.1486
10	113.45	138.3009	21.9048	116.8717	3.0161	113.1498	-0.2646	113.5059	0.0493
FMAPE		4.3996		0.5908		**0.4394**		0.4398	
PMAPE		17.171		2.2029		0.152		**0.0989**	
TMAPE		6.9539		0.9132		0.3819		**0.3716**	
R		0.9525		0.9987		0.9998		**0.9999**	

### Analysis and discussion

**Fig 12:**
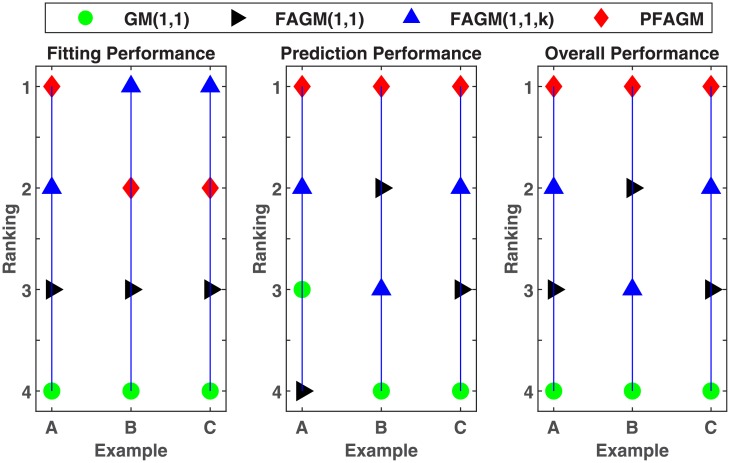
Rankings of different grey models in validations.

According to the results of the above three validation experiments, we can conclude that PFAGM model has a better prediction accuracy than the other three contrast grey model. From an overall perspective, the prediction performances of fractional grey models are superior to the classical GM(1,1) model. In Example A, the MAPE of PFAGM model for fitting is only slightly lower than the other grey models. However, the MAPE of PFAGM for prediction reaches 1.8927%, which is significantly lower than those of the other models. In Example B, the MAPE of PFAGM model for fitting is very closed to the lowest value though it is not the lowest. Moreover, the prediction performance of PFAGM model is slightly better than FAGM(1,1) and FAGM(1,1,k) model, and significantly better than GM(1,1) model. In Example C, PFAGM model obtains the secondary lowest MAPE of fitting which is also very closed to the lowest one among the four models. PFAGM model also achieves the lowest MAPE of prediction. Meanwhile, the overall MAPEs and correlation coefficients of PFAGM model in the three validation are lowest among all grey models. Such indicates that PFAGM model has better stability and adaptability. Fig 12 shows the rankings of various grey models for fitting and prediction performance. It can be clearly noticed that the prediction performance and overall performance of PFAGM is the best in the three validations. Moreover, the nuclear energy consumption of China is used to validate the performance of PFAGM model which obtains better accuracy in Example A. It indicates that PFAGM model can be applied in forecasting renewable energy consumption. In the next section, we apply the novel PFAGM model to predict Chinese wind energy consumption to provide a reference for corresponding decision departments.

## Application

Wind energy is safe, renewable, green, and economical. It is worth developing in all countries all over the world. The13th Five-Year Plan (2016-2020) of China pointed out that the target of total wind installed capacity will be over 210GW at the end of 2020. However, the cumulative wind installed capacity of China has reached 211GW at the end of 2018, according to Global Wind Report 2018 of the GWEC. This means that the target of wind energy was achieved two years ahead of schedule. There is little room for wind energy growth in 2019 and 2020. To provide reference data for government, accurately predicting the wind energy is significant and necessary for formulating or adjusting corresponding wind energy policy. In this section, we focus on forecasting the annual wind energy consumption of China from macro perspectives. Because of small samples and some uncertainty, the grey prediction model is selected to apply in this application. According to the validation of the previous section, PFAGM model has a competitive edge compared with the other three grey models and can be used to forecast wind energy consumption. In this application, we collect the raw wind energy consumption of China from the BP Statistical Review of World Energy 2019. The wind energy consumption from 2009 to 2018, which are tabulated in Table 14, is used to forecast the consumption of the next three years. From Table 14, it can be apparently noticed that wind energy consumption increases rapidly. In 2018, the total wind energy consumption reached 82.8 million tonnes oil equivalent, which is 13.4 times as much as ten years ago. Meanwhile, wind energy is one of the cheapest forms of energy in many countries and is a kind of renewable energy used widely. Therefore, accurately forecasting wind energy consumption plays a vital role in reducing energy expenditure and carbon emissions. Firstly, we partition the raw sequence into two groups to build a model and test the model. The first group, including the consumption from 2009 to 2017, is used to build models for the four grey models separately. The second group, including wind energy consumption in 2018, is used to verify the prediction accuracy of these grey models. The linear parameters of GM(1,1) model can be estimated by using the least-square method. They are -0.22512 and 11.19559. The time response function (TRF) of GM(1,1) model can be represented as:

$$\begin{array}{c} x^{\left(1\right)}\left(k\right)=55.9317e^{-0.2251(k-1)}-49.7317 \end{array}$$ (50)

**Table 14:** Raw data of China’s wind energy consumption (million tonnes oil equivalent).

Year	Wind energy consumption	Year	Wind energy consumption
2009	6.2	2014	35.3
2010	10.1	2015	42
2011	15.9	2016	53.6
2012	21.7	2017	66.8
2013	31.9	2018	82.8

**Fig 13:**
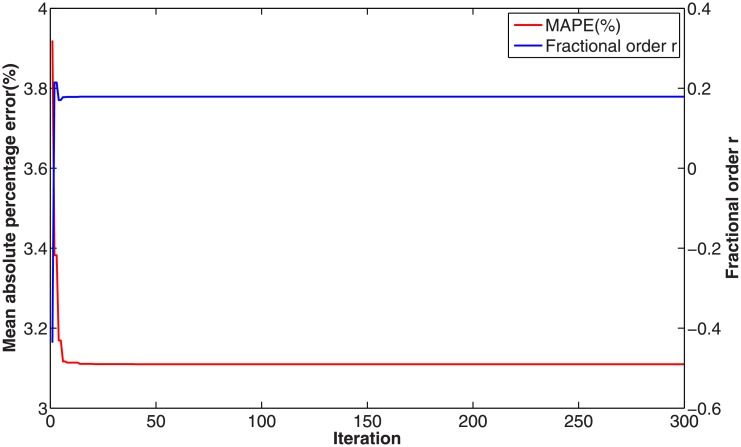
Searching optimal order of PFAGM by using WOA for forecasting Chinese wind energy consumption.

For fractional grey models, their fractional orders should be determined to obtain optimal values. The optimal orders of FAGM(1,1), FAGM(1,1,k), and PFAGM model are searched by using WOA algorithm, which are filled in Table 15. Fig 13 shows than the optimal order of PFAGM model is obtained after a few iterations of WOA algorithm. Then the linear parameters of each fractional grey model can be calculated by using the least-square method, which also are listed in Table 15. The time response function of the three fractional grey models can be respectively represented as follows. The TRF of FAGM(1,1) model is

$$\begin{array}{c} x^{\left(0.36871\right)}\left(k\right)=37.55924e^{0.17072(k-1)}-31.35924. \end{array}$$ (51)

**Table 15:** The optimal parameters of different grey models for forecasting Chinese wind energy consumption.

Parameters	GM(1,1)	FAGM(1,1)	FAGM(1,1,k)	PFAGM
*a*	-0.22512	-0.17072	-0.13851	0.08124
*b*	11.19559	5.35365	4.21723	5.11976
*c*	-	-	3.69298	-0.23365
*γ*	1	0.36871	1.13366	0.17874

The TRF of FAGM(1,1,k) model is

$$\begin{array}{c} x^{\left(1.13366\right)}\left(k\right)=283.1282e^{0.13851(k-1)}-30.4471k-246.4811. \end{array}$$ (52)

The TRF of PFAGM model is

$$\begin{array}{c} x^{\left(0.17874\right)}\left(k\right)=19.6961e^{0.17874k}-14.4727e^{-0.08124(k-1)}-2.8781. \end{array}$$ (53)

**Fig 14:**
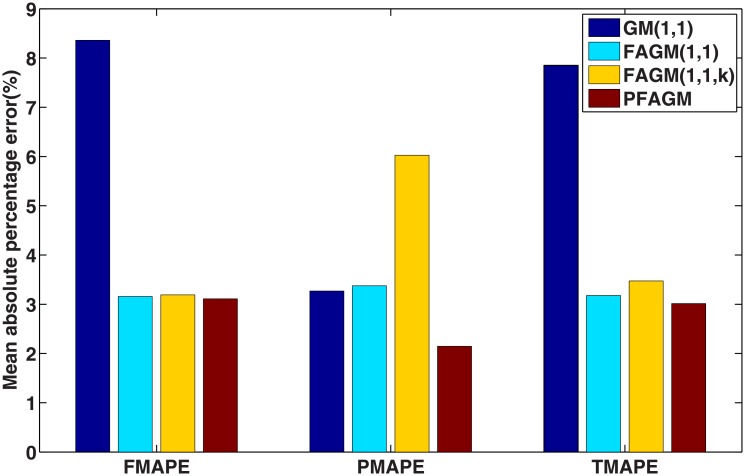
Performance comparison of the proposed and other comparative grey models for forecasting Chinese wind energy consumption.

**Fig 15:**
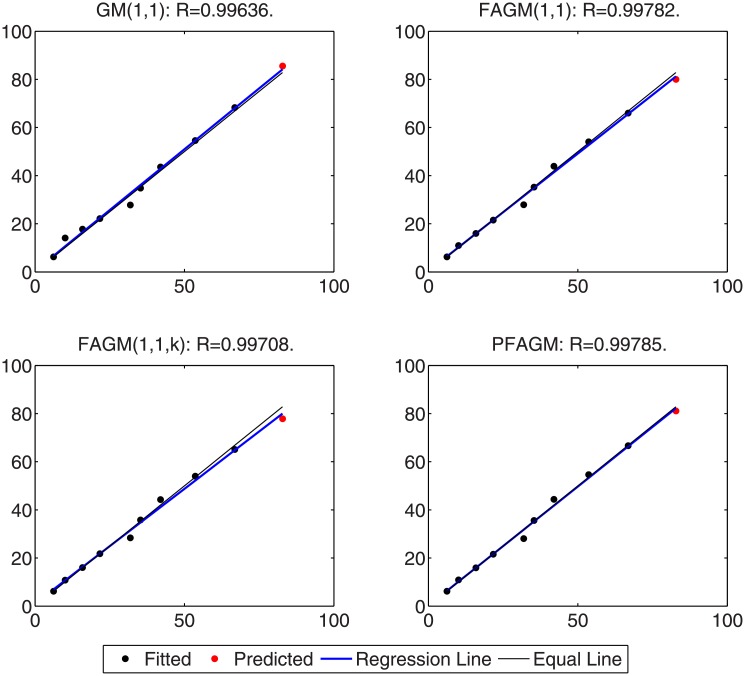
Analysis of detailed results obtained by using the proposed model and other comparative grey models for forecasting Chinese wind energy consumption.

By using inverse fractional or integral order accumulated operation, the restored results of the four grey models can be easily obtained and tabulated in Table 16. From Table 16 and Fig 14, FMAPE, PMAPE and TMAEP of PFAGM model are 3.1100, 2.1456 and 3.0136 respectively while those of GM(1,1) model are 8.3600, 3.2696 and 7.8510, those of FAGM(1,1) model are 3.1559, 3.3757 and 3.1779, and those of FAGM(1,1,k) model are 3.1901, 6.0252 and 3.4736. It can be clearly noticed that the total, fitting and validation MAPE of PFAGM are lowest. Meanwhile, Fig 15 shows that the regression line almost coincides with the equal line. The fitted points of PFAGM model almost are on the regression line, while more fitted points of other grey models deviate from the regression line. R of PFAGM model is also best among the four grey models. Above all, all the above-mentioned evidences suggest that the prediction performance of PFAGM model is better than the other three grey models, and PFAGM model can be used to forecast the wind energy consumption of the future. Then we utilize these established grey models to forecast the wind energy consumption in the next three years. The predicted wind energy consumptions from 2019 to 2021 are tabulated in Table 17. The predicted results of PFAGM model are respectively 98.0472, 118.2918, and 142.4003 in the next three years.

**Table 16:** The results produced by different grey models for Chinese wind energy consumption.

Year	Raw data	GM(1,1)		FAGM(1,1)		FAGM(1,1,k)		PFAGM	
		Value	RPE	Value	RPE	Value	RPE	Value	RPE
2009	6.2	6.2	0	6.2	0	6.2	0	6.2	0
2010	10.1	14.1212	39.8135	10.906	7.9799	10.7861	6.7928	10.8293	7.2208
2011	15.9	17.6863	11.2346	15.9	0	15.9521	0.328	15.9	0
2012	21.7	22.1515	2.0808	21.4735	-1.0437	21.7572	0.2637	21.5801	-0.5525
2013	31.9	27.7441	-13.028	27.8494	-12.6977	28.3244	-11.2087	28.0626	-12.0295
2014	35.3	34.7486	-1.5622	35.2399	-0.1702	35.7865	1.3782	35.5569	0.7278
2015	42	43.5214	3.6225	43.8725	4.4584	44.2901	5.4525	44.3002	5.4767
2016	53.6	54.5092	1.6963	54.0042	0.7541	53.9998	0.746	54.5674	1.8049
2017	66.8	68.271	2.2021	65.9324	-1.2989	65.1026	-2.541	66.6812	-0.1778
2018	82.8	85.5073	3.2696	80.0049	-3.3757	77.8112	-6.0252	81.0234	-2.1456
FMAPE		8.36		3.1559		3.1901		**3.11**	
PMAPE		3.2696		3.3757		6.0252		**2.1456**	
TMAPE		7.851		3.1779		3.4736		**3.0136**	
R		0.9964		0.9978		0.9971		**0.9979**	

**Table 17:** China’s wind energy consumptions from 2019 to 2021 predicted by different grey models.

Year	GM(1,1)	FAGM(1,1)	FAGM(1,1,k)	PFAGM
2019	107.0951	96.6314	92.3688	**98.0472**
2020	134.1331	116.2954	109.054	**118.2918**
2021	167.9974	139.5689	128.1861	**142.4003**

## Conclusion

In this paper, a novel fractional grey model called PFAGM is put forward based on the grey action quantity optimization of the classic fractional grey model with an exponential term. For fractional order accumulated grey models, it is the key to seek out their optimal orders to obtain the best prediction accuracy. In PFAGM model, WOA algorithm is adopted to search its optimal order. Moreover, its linear parameters are estimated by using the least-square method on the basis of the optimal order. Meanwhile, there are some various structures of the grey model behind PFAGM model, which can be reduced into GM(1,1) and FAGM(1,1) easily. It maybe indicates that PFAGM model has more predictive power than traditional grey models. The results of validation experiments on real-life datasets show that PFAGM model has better prediction performance than the other three grey models. So PFAGM model is used to predict Chinese wind energy consumption. The predicted values of GM(1,1) in the next 3 years are 107.0951, 134.1331, 167.9974 respectively while those of FAGM(1,1) are 96.6314, 116.2954, 139.5689 and those of FAGM(1,1,k) are 92.3688, 109.0540, 128.1861 respectively. It indicates that the results of GM(1,1), FAGM(1,1,k) maybe have more some deviation from the truth. Results of PFAGM model are in the middle of the other grey models’ results. So, its predicted results can be as reference data of adjusting wind energy policy. Above all, it can be drawn that PFAGM model is efficient to realize short term prediction for time series, especially the sequence with small samples or uncertainty. In the future, the novel PFAGM model can be applied in more applications such as carbon emission forecasting, management of the petroleum reservoirs and so on.
